# Large-area metal-integrated grating electrode achieving near 100% infrared transmission

**DOI:** 10.1038/s41377-026-02270-0

**Published:** 2026-04-10

**Authors:** Karolina Bogdanowicz, Weronika Głowadzka, Tristan Smołka, Michał Rygała, Marcin Kałuża, Marek Ekielski, Oskar Sadowski, Magdalena Zadura, Magdalena Marciniak, Marcin Gębski, Michał Wasiak, Marcin Motyka, Anna Szerling, Tomasz Czyszanowski

**Affiliations:** 1 Photonics Group, Institute of Physics, Lodz University of Technology, ul. Wólczanska 219, Łódź, Poland; 2 Łukasiewicz Research Network - Institute of Microelectronics and Photonics, al. Lotników 32/46, Warsaw, Poland; 3 Laboratory for Optical Spectroscopy of Nanostructures, Department of Experimental Physics, Faculty of Fundamental Problems of Technology, Wrocław University of Science and Technology, Wybrzeże Wyspiańskiego 27, Wrocław, Poland; 4 Institute of Electronics, Lodz University of Technology, al. Politechniki 8, Łódź, Poland; 5 Institute of Microelectronics and Optoelectronics, Warsaw University of Technology, Koszykowa 75, Warsaw, Poland

**Keywords:** Mid-infrared photonics, Optoelectronic devices and components, Metamaterials, Nanophotonics and plasmonics

## Abstract

Highly transparent and conductive electrodes operating in the infrared (IR) are critically needed for a broad range of technologies, including light-emitting diodes, lasers and photodetectors, which are key building blocks of infrared cameras, LiDARs, and thermal systems such as IR heaters. While transparent conductive electrodes (TCEs) have seen substantial progress in the visible spectrum, their performance in the IR remains limited due to increased absorption and reflection caused by the plasma resonance of free carriers in conductive materials. Here, we demonstrate a large-area TCE based on a metal-integrated monolithic high-contrast grating (metalMHCG) fabricated on a GaAs substrate. This structure acts as an effective antireflection coating, achieving near-unity transmission of unpolarized mid- to far-infrared (M-FIR) light. The metalMHCG exhibits 94% transmission at a wavelength of 7 μm, corresponding to 135% relative to transmission through a flat GaAs–air interface, while maintaining an exceptionally low sheet resistance of 2.8 Ωsq^−1^. By simultaneously delivering excellent optical transparency and electrical conductivity, the metalMHCG establishes a new performance benchmark among M-FIR TCEs and provides a versatile platform for next-generation high-power optoelectronic devices.

## Abstract (web summary)

Demonstration of a transparent conductive infrared electrode enabling nearly absorption- and reflection-free transmission with metal-like conductivity.

## Graphical abstract

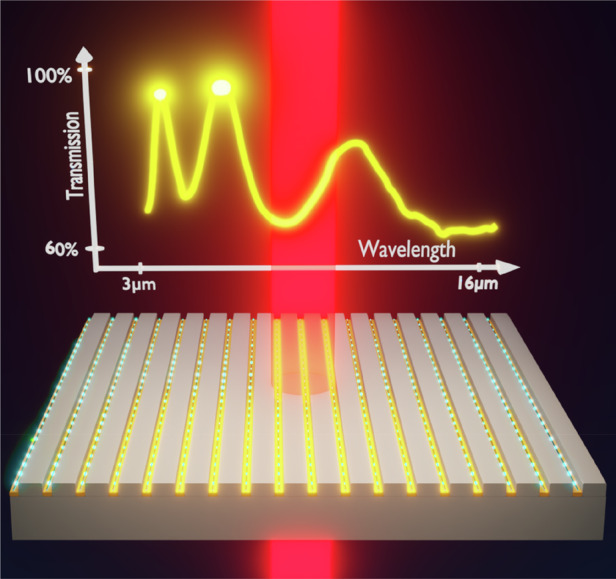

## Introduction

Transparent conductive electrodes (TCEs) aim to balance two conflicting properties: high optical transparency and excellent electrical conductivity. Achieving high conductivity in TCEs requires a high concentration of free carriers in the TCE material, which inherently limits their transmittance. This fundamental trade-off between electrical conductivity and light transmission has been extensively studied in TCEs for visible spectrum (VIS) and shortwavevelength infrared (SWIR) applications, driving significant advancements in the field. As a result, TCEs presently play a crucial role in a wide range of optoelectronic devices, including sensors^1^, displays^2^, light-emitting diodes^3–5^, photovoltaics^6,7^, and flexible transparent electronics^8^.

Among the various methods for implementing TCEs, the use of indium tin oxide (ITO) is the most common^9^. Despite mass scale production of ITO-based TCEs, its replacement is anticipated in the near future due to the scarcity of indium. Therefore, numerous approaches based on other transparent conductive oxides (TCO), graphene, thin metal plates or metal networks, and many more are investigated^10^.

In the mid-to-far infrared (M-FIR) spectral range, the primary application of TCEs is their integration with optoelectronic devices such as light emitting diodes (LEDs), photodetectors (PDs) and lasers. Additionally, TCEs can serve as electromagnetic shields for M-FIR optoelectronic devices^11^, enhance the performance of transparent heaters^12^ and improve the functionality of liquid crystal optical switches^13^. This integration of TCEs with optoelectronic devices aims to increase the performance of the devices, but poses fundamental challenges. The main challenge arises from the resonance frequency of the free electron plasma in conductors (metals, nanocarbons, conductive oxides), which is located in the infrared spectrum^14,15^. Enhanced interaction of the electromagnetic field with free electrons results in high absorption and reflection in all conductive materials. In the case of conductive oxides that are transparent in VIS such as ITO, In_2_O_3_, CuScO_2_ and many more, lattice vibrations and impurity scattering further contribute to increased infrared absorption and a reduction in electrical conductivity, thereby limiting their practical use in infrared applications^16^.

In most TCE demonstrations, transmittance is typically defined as the percentage of light propagating through the TCE layer alone. However, when a TCE is deposited on a high-refractive-index substrate, reflection at the substrate surface becomes a non-negligible factor that further reduces overall transmission. It seems inevitable that due to the intrinsic absorption of TCEs their implementation on the surface of the substrate reduces transmission compared to the case of bare substrate–air interface. In this work the transmission through the bare interface is referred to as the Fresnel limit (*T*_Fr_) that can be calculated using the Fresnel formula:

1 $${T}_{{Fr}}=1-{\left(\frac{{n}_{s}-{n}_{a}}{{n}_{s}+{n}_{a}}\right)}^{2}=\frac{4{n}_{s}{n}_{a}}{{\left({n}_{s}+{n}_{a}\right)}^{2}}$$

where *n*_s_ and *n*_a_ are the refractive indices of the substrate and air, respectively, and the absorption of the substrate is assumed to be negligibly small. In the case of TCEs deposited on semiconductor devices, the Fresnel limit is typically less than 80% in the case of wide bandgap semiconductors and less than 70% in the case of narrow bandgap semiconductors. Surpassing the Fresnel limit remains a significant challenge for TCEs operating in the VIS and SWIR spectral ranges and is generally considered unattainable in the M-FIR regime. Notable exceptions include ultrathin-metal-film-based TCE operating in the VIS, which were shown by C. Ji et al.^17^ to exceed the Fresnel limit by 0.3%, as well as an ultrathin silver film combined with a high-index overlayer reported by Ryu et al.^18^, achieving relative transmittance with respect to the Fresnel limit (*T*_R_) of 123% at 1300 nm through a destructive-interference design, together with a sheet resistance of 14 Ωsq^−1^.

In our previous studies, we demonstrated that various configurations of monolithic high-contrast gratings integrated with metal (metalMHCGs) can function as efficient transparent conducting electrodes (TCEs), enabling nearly 100% transmission (*T*_R_ > 130%) of infrared light polarized both parallel and perpendicular to the metalMHCG stripes^19,20^. Furthermore, in^21^, a metalMHCG structure fabricated on GaAs was designed to maximize the transmission of light polarized parallel to the metal stripes, achieving a value of 92% (*T*_R_ = 133%) as a result of a low-quality-factor Fabry–Pérot (F–P) resonance. Simultaneously, this configuration exhibited transmission exceeding 50% for the orthogonal polarization, yielding an overall transmission of approximately 75% for unpolarized light and surpassing the Fresnel limit at the GaAs–air interface by 6% at a wavelength of 9 μm. Such high transmission is realized within the first transmission band (see Fig. 2a and the corresponding discussion). In the present study, we advance both the design and fabrication of metalMHCGs, introducing an approach that enables near-perfect transparency to unpolarized M-FIR radiation over centimeter-scale areas while maintaining record-low sheet resistance. In the proposed configuration, the metalMHCG architecture decouples carrier transport from optical transmission, thereby minimizing infrared absorption and substantially enhancing transparency through the simultaneous formation of F–P resonances for both orthogonal polarizations in the third transmission band (see Fig. 2a and the corresponding discussion). Importantly, the metalMHCG exhibits properties similar to those of anti-reflective layers, ensuring that its effective refractive index, as experienced by the interacting radiation, remains lower than that of the substrate, as in the case of conventional anti-reflective layers.

In the presented experimental demonstration, the metalMHCG consists of gold stripes embedded between periodically distributed GaAs stripes, structured on top of a GaAs substrate. This configuration achieves a transmission of 94% for unpolarized light at a central wavelength of 7 μm and results in a record-high relative transmittance of 135% with respect to the Fresnel limit. Additionally, the transmission bandwidth exceeding the Fresnel limit is 1.5 μm, corresponding to a relative bandwidth of 21%.

## Results

### Configuration and simulations

**Fig. 1:**
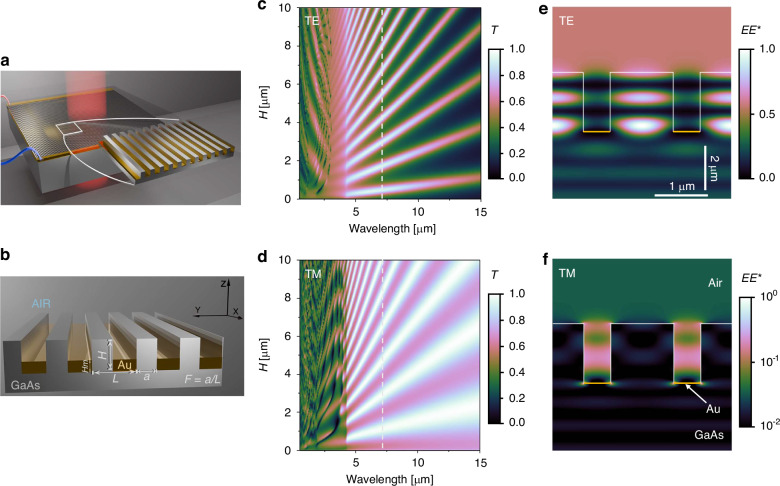
Monolithic high-contrast grating integrated with metal: structure and optical response. **a** Conceptual visualization of the metalMHCG composed of a one-dimensional grating on a GaAs wafer with gold stripes implemented in the grooves between the semiconductor stripes; **b** Zoomed slice of the metalMHCG indicating the cross-section of the configuration, with geometrical definition of the grating parameters and the coordinate system. Calculated transmission (*T*) maps of the metalMHCG under normal incidence of **c** TE and **d** TM polarized light in the domain of the wavelength, and height of the semiconductor stripes (*H*) for *L* = 1.4 μm, *F* = 0.74, *H*_m_ = 50 nm. The white dashed lines in both figures indicate the wavelength of 7 μm. Light intensity (EE*) distribution under normal incidence from the substrate side in the case of **e** TE and **f** TM polarization in the yz-plane of the metalMHCG cross-section

To demonstrate the transmission properties of metalMHCG using numerical methods, we consider the structure illustrated in Figs. 1a, b, along with the Cartesian coordinate system adopted in analysis. The structure used in the calculations consists of a semi-infinite GaAs substrate, above which is a semi-infinite air superstrate. The surface of the GaAs in the *xy*-plane is patterned into an infinite one-dimensional grating consisting of parallel rectangular stripes along the *x*-direction, with alternating GaAs and gold stripes, both with a rectangular cross-section (Fig. 1a). The height of the GaAs stripes (*H*) exceeds that of the gold stripes (*H*_m_). Additional parameters of the metalMHCG, also illustrated in Fig. 1b, include the period (*L*), the width of the semiconductor stripes (*a*), and their ratio, referred to as the fill factor (*F*). Light polarized along the *x*-direction (parallel to the stripes) is referred to as transverse-electric (TE) polarization while orthogonal polarization is referred to as transverse-magnetic (TM).

In the numerical model, we utilize the plane wave admittance method^22^ we used previously^19–21^, showing consistency with experimental results. The simulation considers the cross-section of the structure in the *yz*-plane, as illustrated in Fig. 1b. In the neglected *x*-direction, the solution is assumed to be a plane wave, corresponding to a plane wave incident normally on the metalMHCG surface. In the *y*-direction, we consider a single period of the grating with periodic boundary conditions, which extends the metalMHCG to infinity in this direction. We determine transmission for the case where light propagates perpendicularly to the metalMHCG plane from the substrate side to the air. The opposite propagation direction yields the same result.

In what follows, we consider an example illustrating an optimized structure that enables maximum transmission of unpolarized light for a wavelength of approximately *λ*_max_ = 7 μm. The dimensions of the metalMHCG stripes, as defined in Fig. 1b, are *L* = 1.4 μm, *F* = 0.74, *H*_m_ = 50 nm. The dispersion of the refractive indices of GaAs (*n*_GaAs_) and Au (*n*_Au_) follow the experimental dependencies^23,24^. The optimization procedure for different refractive indices of the metalMHCG is detailed in^25^.

Figure 1c, d present numerically calculated transmission spectra as functions of the semiconductor stripe height (*H*) and the wavelength (*λ*) for TE and TM polarization, respectively. The maps exhibit two wavelength regions with noticeably different transmission properties. In the wavelength range *λ* < *n*_GaAs_*L* ≈ 4.5 μm, the transmission is the result of two modes interacting within the subwavelength grating, in what we refer to as the subwavelength region, as has been thoroughly discussed in^26^. In this region, only the zero-order diffraction of the grating can propagate in air, while more than one diffraction order can propagate in GaAs. Within this spectral range, the transmission through the metalMHCG can reach significant values, as indicated by the brighter areas in the transmission maps for both polarizations. However the spectral width of transmission above 70% remains narrow.

For *λ* > *n*_GaAs_*L*, the metalMHCG transmits and reflects only the zeroth diffraction order under normal incidence. In this spectral range, the metalMHCG exhibits properties typically observed in metastructures, characterized by an averaged interaction of both orthogonal polarizations with the structure^27^. We will refer to this region as the deep subwavelength region, which is the primary focus of this article. In this region, high-transmission bands exist for both polarizations, which are significantly broader spectrally than the high-transmission regions present in the subwavelength region.

Analysis of Fig. 1c, d, along with Supplementary Fig. S1, indicates that both orthogonal polarizations display a typical etalon-like transmission dependence on the semiconductor stripe height (*H*). This behavior suggests that the metalMHCG functions as an etalon (Fabry-Perot (F-P) resonator) formed between the metalMHCG-air interface on one side and the metalMHCG–GaAs interface on the other, where the homogeneous GaAs substrate has a refractive index of 3.29 at a wavelength of 7 μm^28^.

Based on the periodicity of transmission oscillations with respect to *H* for both polarizations at a wavelength of *λ* = 7 μm (see Fig. S2 in the Supplementary Information), one can determine the effective refractive indices of the metalMHCG, which are *n*_effTE_ = 2.95 and *n*_neffTM_ = 2.11 for TE and TM polarizations, respectively. The different periodicities for the two polarizations and hence different effective refractive indices result from different spatial field distributions of TE and TM polarizations within the metalMHCG. Figures 1e, f indicate that TE polarization is mainly confined within the semiconductor stripes, whereas TM polarization is confined in the air gaps between the stripes. Additionally, the optical field of both polarizations penetrates the metal stripes only to a limited extent, as demonstrated in Fig. S1 in the Supplementary Information. Plasmonic effects at the GaAs-gold interface for TM polarization are almost entirely suppressed compared to similar structures composed solely of parallel metal stripes^29^. Interestingly, in the MHCG without metal, it is impossible to achieve such high transmission as can be obtained in the metalMHCG (for unpolarized light)^25^. A comparison of transmission through the metalMHCG and MHCG can be found in Fig. S3 of the Supplementary Information.

The periodic behaviour of transmission with respect to the height of the metalMHCG stripes, which differs for each polarization, enables an optimal *H* to be found at which both polarizations achieve nearly 100% transmission, resulting in almost complete transmission of unpolarized light. This condition can be satisfied with relatively small *H* by tuning *F*, which affects the effective refractive indices of both polarizations.

**Fig. 2:**
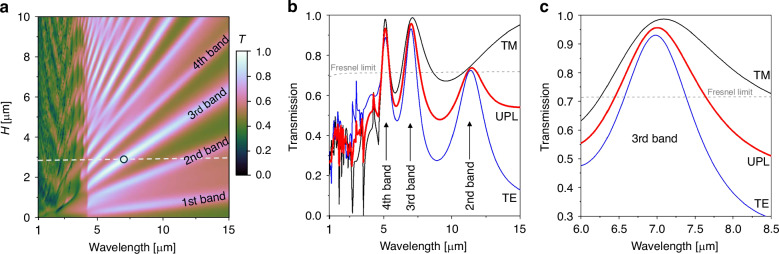
Calculated transmission properties of the metalMHCG under normal incidence. **a** Calculated transmission (*T*) map of the metalMHCG under normal incidence of unpolarized light in the domain of the wavelength, and height of the semiconductor stripes (*H*) for *L* = 1.4 μm, *F* = 0.74, *H*_m_ = 50 nm, where the white dashed line indicates *H* = 2.79 μm and black circle indicates the global maximum of the transmission at the wavelength of 7 μm; **b** calculated transmission spectra of TE (blue) and TM (black) polarizations as well as unpolarized (red) light along the white dashed line from **a**; **c** zoom of the spectrum in the proximity of transmission maximum. The Fresnel limit indicates transmission through the plane interface between GaAs and air in **b** and **c**

Figure 2a presents the transmission map of unpolarized light through the metalMHCG. The high-transmission bands, which form regular regions in the (*H, λ*) domain for polarized light (see Fig. 1c, d), transform to a more complex pattern for unpolarized light due to the different effective refractive indices of the two polarizations. Consequently, the transmission of unpolarized light exhibits non-periodic behavior. The successive high-transmission bands of unpolarized light, occurring with increasing *H*, exhibit transmission maxima of different values.

Figure 2b presents the transmission spectrum of unpolarized light (UPL) for the optimized configuration defined by the parameters *L* = 1.431 μm, *F* = 0.734, *H* = 2.787 μm, and *H*_m_ = 50 nm, in which the second, third, and fourth transmission bands are indicated. Within the third transmission band, the global transmission maximum reaches *T*_max_^(3)^ = 95.7%, while transmission exceeding the Fresnel threshold (*Δλ*_Fr_^*(*3)^) spans over 1.2 μm, corresponding to a relative spectral width (*Δλ*_Fr_^(3)^/*λ*_max_^(3)^) of more than 17.2%. In the fourth transmission band, these values are *T*_max_^(4)^ = 93.4%, *Δλ*_Fr_^(4)^/*λ*_max_^(4)^ = 12.5%, and in the second transmission band *T*_max_^(2)^ = 73.7%, *Δλ*_Fr_^(2)^ /*λ*_max_^(2)^ = 5.0%.

The maximum transmission is achieved with slightly different peak values for TE and TM polarizations, as shown in Fig. 2c. While equalizing the transmission of both polarizations is possible by adjusting the metalMHCG parameters, it comes at the cost of a 3% reduction in total transmission in the analyzed case. A more comprehensive numerical analysis of the optical properties, including the angular dependence of transmission, the impact of the thickness of the metal on transmission, and the tunability characteristics, is presented in Section S1 of the Supplementary Information and in^25^.

The metalMHCG reveals superb electrical properties as volume of the metal in metalMHCG is significantly larger than in any other TCE used in any spectral range. Considering the geometric parameters of the gold stripes (*H*_m_ = 50 nm, *L-a* = 364 nm) and assuming a bulk gold resistivity of (2.44 × 10^−8^ Ωm) the optimal metalMHCG achieves a sheet resistance of 2 Ωsq^-1^.

### Fabrication

**Fig. 3:**
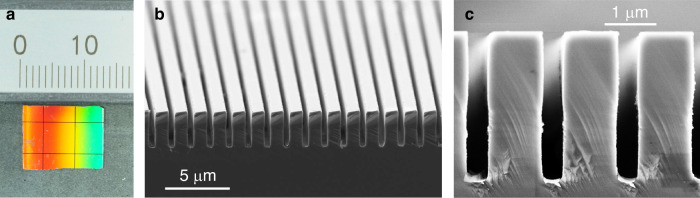
Optical and scanning electron microscope (SEM) images of the fabricated metalMHCG. **a** Visible light image of the sample, black lines are spacers between metalMHCGs patches, **b** and **c** cross-section SEM images of metalMHCG in various magnifications. Metal stripes visible in bottom of the grooves in **c**

The fabricated metalMHCG covers more than 1 cm^2^ of a GaAs wafer, in the form of nine patches. The central square-shape patch has a side length of 5×5 mm (Fig. 3a). The nominal geometric parameters of the realized structure are the same as those of the metalMHCG structure used in the numerical analysis. An anti-reflection coating is not deposited on the opposite wafer surface, due to the significant infrared absorption of the dielectric materials.

The realization of the GaAs-based metalMHCG with gold stripes involves a combination of plasma-enhanced chemical vapor deposition (PECVD), electron beam lithography (EBL), inductively coupled plasma-reactive ion etching (ICP-RIE), and e-beam physical vapor deposition (EBPVD). The two main technological challenges are the fabrication of a GaAs grating with parallel grooves with an aspect ratio of depth to width of 7.7 and the accurate placement of metal stripes at the bottom of these grooves (see Fig. 3c).

The actual cross-section shape and dimensions of the processed metalMHCG were determined based on SEM images: *L* = 1469 nm with standard deviation of 2 nm, *F* = 0.747 with standard deviation of 0.007, *H* = 2890 nm with standard deviation of 40 nm, *H*_m_ = 51 nm with standard deviation of 2 nm. A detailed statistical analysis of fabrication uniformity is presented in Section S2 of the Supplementary Information.

### Optical and electrical properties

**Fig. 4:**
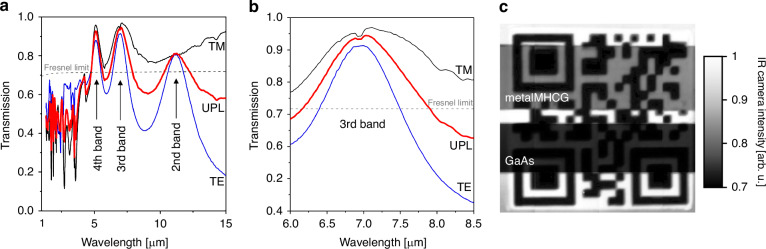
Experimental transmission characteristics and infrared imaging of the metalMHCG. **a** Experimental transmission spectra of the metalMHCG in the case of TE (blue) and TM (black) polarizations as well as unpolarized (red) light; **b** zoom of the experimental spectrum in the proximity of the transmission maximum in the third band. Fresnel limit indicates transmission through plane interface between GaAs and air; **c** infrared image taken in the wavelength range of 4.8–5.0 μm (fourth band), showing a QR code heated to 80 ^◦^C in the background and two samples from the same GaAs wafer: metalMHCG on the GaAs substrate (metalMHCG) and bare GaAs substrate (GaAs) placed above the QR code

The experimentally measured transmission spectrum (Fig. 4) closely aligns with the numerically computed spectrum (see Fig. S5a and Sections S3 and S4 in Supplementary Information). In the deep subwavelength regime, three distinct transmission maxima for unpolarized light are observed, corresponding to the second, third, and fourth transmission bands, with peak wavelengths at 5.1 μm, 7.1 μm, and 11.2 μm and maximal transmission of 92.5%, 94%, and 81%, respectively. Within the subwavelength range (*λ* < 4.5 μm), multiple narrow spectral regions exhibit pronounced reflection and transmission features, which, according to numerical simulations, can exceed 90% within a very narrow bandwidth. However, in the specific configuration studied here, the transmission maxima in this range remain below the Fresnel limit. In the long-wavelength region near 15 μm, the experimentally measured transmission reaches approximately 60%, which aligns well with numerical simulations. According to these calculations, at even longer wavelengths beyond the experimental range the transmission remains relatively stable, at around 50–60%. Details of the measurement uncertainty analysis are provided in Section S5 of the Supplementary Information.

The primary goal in designing the metalMHCG structure was to achieve the highest possible transmission, positioned arbitrarily within the M-FIR range, with a spectral bandwidth comparable to the emission or absorption linewidths of optoelectronic devices operating in this region. Consequently, our analysis focuses on the third transmission band, which aligns with these objectives and exhibits a global transmission maximum of 94% for the given configuration. The experimentally determined maximum transmission is only slightly lower than the theoretical prediction of 95.7%. For TM polarization, the transmission reaches 96.9%, surpassing the TE polarization transmission, which reaches 91.3%. The peak unpolarized light transmission, normalized to the Fresnel level (relative transmission), reaches 135% for the analyzed metalMHCG—significantly exceeding previously reported maximum values^17,21^. The spectral width of unpolarized light transmission exceeding the Fresnel limit is approximately 1.5 μm, corresponding to a relative spectral bandwidth of 21% with respect to the peak wavelength. The broader transmission spectrum compared to the numerical results presented in Section “Configuration and simulations” is attributed to the non-rectangular cross-section of the stripes, which enhances the transmission bandwidth. The experimental and numerical spectra for the actual metalMHCG cross-section are compared in Section S4 of the Supplementary Information.

To evaluate the transmission properties of the metalMHCG, we performed infrared imaging using a cooled InSb infrared camera equipped with a 1/4 inch extension ring to enhance magnification^30^ and a 4.8–5.0 μm bandpass optical filter, selected to match the spectral region of the fourth transmission band of the metalMHCG. Figure 4c shows a thermographic image of a QR code patterned with FR4 laminate on copper cladding at a temperature of 80 °C. The horizontal, darker, rectangle-shaped regions correspond to areas imaged through a bare GaAs wafer (bottom sample) and through the GaAs wafer with the metalMHCG structure deposited on one of its interfaces (top sample). In both samples, light is reflected by the flat bottom GaAs–air interface. The difference in brightness indicates higher transmission through the opposite interface with the metalMHCG (top sample) compared to the flat GaAs–air interface (bottom sample). Details of the experimental setup and imaging procedure are provided in Section S6 of the Supplementary Information.

To accurately evaluate the sheet resistance of the metalMHCG structure, an indirect approach is required. Due to the inherently low resistance of the fabricated large-area metalMHCG, its total resistance is significantly lower than the parasitic resistance introduced by the electrical contacts and probing system, as confirmed by short-circuit configuration measurements. Since the total resistance of the metalMHCG is primarily governed by its metallic components, we evaluate the sheet resistance using dedicated test structures. These structures consist of a reduced number of parallel gold stripes, allowing the overall resistance to reach a level that is measurable with standard laboratory equipment while minimizing errors associated with the parasitic resistance of the measurement setup.

The test structures were fabricated on a non-conductive silicon substrate and include configurations with 4, 6, 8, and 12 gold wires, connected in parallel, each 2 mm in length and with nominal dimensions of 50 nm in height and 365 nm in width. These dimensions are identical to those used in the metalMHCG. Each configuration was replicated four times (Fig. S13a–c in the Supplementary Information).

The current–voltage measurements exhibited linear behavior, allowing the electrical conductivity of a single gold stripe to be determined as approximately 0.25 mS (Fig. S13d in the Supplementary Information). Based on the stripe dimensions and the metalMHCG period, the corresponding sheet resistance is estimated to be around 2.8 Ωsq^−1^. This value is only about 40% higher than the theoretical sheet resistance calculated using the bulk conductivity of gold, which does not account for size effects in thin metallic wires or the presence of impurities. The electrical properties of the fabricated metalMHCG are consistent with those reported for a previously demonstrated metalMHCG configuration designed for polarized light^21^, which further indicates that a noticeable increase in sheet resistance occurs only for current densities exceeding 20 MA cm^−2^ in metalMHCG. Such current densities are typically required for quantum cascade lasers to achieve active-region current densities above 10 kA cm^−2^, which are among the highest reported for infrared optoelectronic devices.

## Discussion

**Fig. 5:**
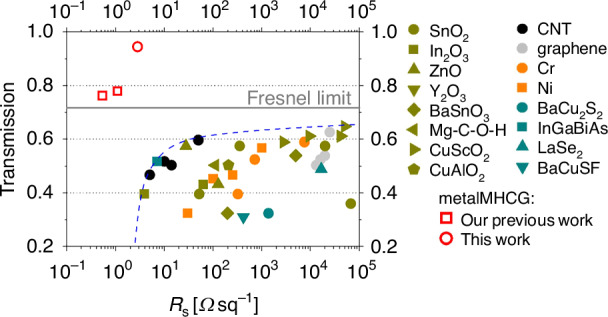
Optical maximal transmissions at M-FIR as a function of sheet resistance for TCEs based on various material approaches: (SnO_2_^31–34^, In_2_O_3_^35,36^, ZnO^37,38^, Y_2_O_3_^39^, BaSnO_3_^40^, Mg-C-O-H^41^, CuScO_2_^42,43^, CuAlO_2_^44^, carbon nanotube (CNT)^45^, graphene^46^, Cr^47^, Ni^47^, BaCu_2_S_2_^48^, InGaBiAs^49^, LaSe_2_^50^, BaCuSF^51^, metalMHCG our previous work^21^). The reported transmittance has been recalculated to represent the transmission through the TCE deposited on a GaAs substrate, the grey dashed line presents transmission Fresnel limit at 7 μm. The red circle represents unpolarized transmission near 7 μm for the metalMHCG presented in this work. The numerical values of the data points are summarized in a Table S4 in the Supplementary Information

We compare the properties of the metalMHCG with other TCEs operating in the mid-infrared range by evaluating their optical and electrical characteristics, as reported in references^31–51^. In most cases, the transmittance or relative transmission of these TCEs was assessed in configurations where they were implemented on low-refractive-index substrates such as glass or polymers. For the purpose of a consistent comparison, the transmission values of the TCEs were recalculated assuming their integration on a GaAs substrate with a representative refractive index of 3.29, for which the corresponding Fresnel limit is 71.5%. Figure 5 presents various TCEs as data points in the space defined by maximum transmission and sheet resistance. The distribution of the points corresponding to the most efficient configurations, excluding the red-marked data points representing our previous and current work, follows a trend indicated by the blue dashed line. This trend shows that achieving transmission exceeding 60% (corresponding to a transmission relative to the Fresnel limit *T*_R_ > 80%) is typically possible only for devices with a sheet resistance greater than 50 Ωsq^−1^. A notable example is a 200 nm-thick CuSCO_2_ layer^43^, which exhibits the highest reported transmission of 64% (*T*_R_ = 90%) and sheet resistance of 50 kΩsq^−1^. At the opposite end of the trade-off line, a 1.3 μm-thick In_2_O_3_ layer^35^ demonstrates the lowest reported sheet resistance of 3.8 Ωsq^−1^, accompanied by a transmission below 40% (*T*_R_ = 55%). Among the considered TCEs, a 100 nm-thick carbon nanotube (CNT)-based layer^45^ offers an attractive balance between optical and electrical performance, with a transmission of 55% (*T*_R_ = 83%) and a sheet resistance of 50 Ωsq^−1^.

MetalMHCGs are the only TCEs that surpass the Fresnel limit and are positioned significantly above the trend line. Our previous work^21^, which demonstrated a metalMHCGs operating in the first transmission band (see Fig. 5a) and optimized for efficient transmission of polarized radiation, are marked in the figure as a red hollow squares. This configuration achieved a record-low sheet resistance of 0.5 Ω sq^−1^ and an unpolarized light transmission of 73% (*T*_R_ = 106%). The TCE presented in this work, indicated by the red circle, outperforms all non-metalMHCG TCEs in both parameters simultanously, offering 1.7 times higher transmission and 30% lower sheet resistance than any best performing TCEs in either category. While the transmission of metalMHCG exhibits greater spectral variability than that of conventional TCEs—due to its reliance on a low quality-factor resonance—its spectral width is typically broader than the emission spectra of IR light sources such as LEDs or lasers, and it can be arbitrarily positioned within the M-FIR spectrum by design (see Section S1 D and Fig. S6 in Supplementary Information).

TCEs operating in the M-FIR face at least two key challenges compared to those designed for the visible and SWIR ranges. The proximity to the plasma frequency of free electrons leads to significantly higher absorption, and integration with emitters such as quantum cascade lasers or interband cascade LEDs and lasers imposes the need to sustain very high current densities without excessive power dissipation or performance degradation. These devices require the highest current densities among all optoelectronic systems. As a result, M-FIR TCEs must achieve a sheet resistance below 10 Ω sq^−1^, an order of magnitude lower than their visible-range counterparts^52^.

Configurations based on metalMHCGs can be envisioned to be monolithically integrated as top-surface layers in planar optoelectronic devices using standard III–V semiconductor processing. Such implementations are expected to enable uniform current injection and efficient light extraction in lasers and LEDs. In photodetectors, they may serve as a transparent top electrode and are anticipated to support fast carrier collection and high-speed operation without perturbing the optical field distribution in the active region. Only minor design modifications are required to ensure efficient electrical coupling between the metal and the semiconductor without causing any significant degradation of optical transmission^20^. Furthermore, wafer-level fabrication of the metalMHCG is expected to be feasible using established industrial lithography platforms. For feature sizes above 100 nm, mature deep-UV (193 nm wavelength) immersion photolithography with multiple patterning^53,54^, as well as nanoimprint lithography^55,56^, provide scalable and high-throughput fabrication routes. For feature sizes above 200 nm, stepper-based optical lithography enables efficient wafer-scale processing and large-area fabrication^57^. These approaches are also compatible with standard III–V semiconductor processing, supporting reliable industrial-scale implementation. Taken together, the record-low sheet resistance combined with exceptionally high optical transmittance positions metalMHCGs as a leading candidate for transparent conductive electrodes in high-power optoelectronic applications. In photodetectors employed in optical communication, imaging systems, laser guidance, and biosensing, such highly transparent electrodes enhance carrier collection efficiency and reduce response times by enabling faster carrier transport from the active region to the electrode^18^. Additionally, metalMHCGs are suitable for shielding applications in aviation, military, and medical fields. They can protect sensitive electronics from electromagnetic fields at wavelengths longer than IR, due to their dense metal distribution, while preserving image clarity in the infrared range due to their deep-subwavelength geometry. Finally, metalMHCGs are well-suited for use as transparent heaters in energy-efficient heating applications and as infrared liquid crystal optical switches for advanced light modulation technologies. The proposed metalMHCG exceeded the Fresnel limit, which had never been significantly surpassed before, and approached total transmission, clearly outperforming all previously proposed solutions and setting a new performance benchmark.

## Methods

### Fabrication

High aspect ratio metalMHCG TCE was created by transferring the pattern of the grating generated in the EBL process from resist to GaAs substrate through three layers of hard masks consisting of SiO_2_/Cr/SiO_2_ in the ICP-RIE plasma etching procedure. The pattern was transferred to the substrate using BCl_3_/N_2_ in short cycles of etching and cooling, achieving smooth, slightly concave grating stripes profile. This profile prevents the deposition of metal from deposition on side walls of the groves, which could potentially contribute to absorption. This shape allowed for precise gold deposition on the bottom of the grooves and easy removal of the leftover hard mask in the buffered hydrofluoric acid solution. The embedded gold stripes are expected to exhibit long-term stability comparable to conventional Au-based metallizations in arsenide-based devices, which routinely operate for thousands of hours under repeated thermal cycling. Mechanical confinement within the GaAs grating walls, together with thin adhesion or diffusion-barrier layers (e.g., Ti, Pt, Ni), limits gold diffusion^58^ while preserving optical and electrical performance^20^. The small thermal expansion mismatch between Au and GaAs^59,60^ further reduces thermomechanical stress. Although long-term aging tests remain beyond the scope of this work, no degradation was observed under the elevated temperatures used in our experiments, indicating robust thermal and structural stability.

### Measurements

Transmission measurements were conducted using a Vertex 80v vacuum Fourier Transform Infrared spectrometer (FTIR) from Bruker. The light was generated by a polychromatic source (either a halogen lamp or a Globar, depending on the spectral range) and focused by an optical system with parabolic mirrors onto the metalMHCG at a normal incident angle, creating a roughly 1-mm diameter spot entirely contained within the 5×5 mm metalMHCG area. The spot size causes the transmission measurement to be averaged over more than 700 periods of the metalMHCG, which is therefore subject to statistical error due to fabrication imperfections, such as the waviness of the semiconductor stripes, inhomogeneity, and discontinuities in the gold stripes. The light intensity was measured using a HgCdTe (MCT) liquid-nitrogen cooled detector. As references, transmission spectra were recorded for an empty chamber and for a piece of the same wafer without a metalMHCG. This approach enabled the measurement of light transmission while eliminating the influence of potential absorption and scattering in the wafer, as well as Fresnel reflection from the wafer’s opposite surface. A detailed setup description can be found in Section S3 of the Supplementary Information.

## Supplementary information

suplement

## Data Availability

Relevant data supporting the key findings of this study are available within the article and the Supplementary Information file. All raw data generated during the current study are available from the corresponding author upon reasonable request.
