# Supplementary material for: Large-area metal-integrated grating electrode achieving near 100% infrared transmission
Source: Light Sci Appl. 2026 Apr 10;15:195. doi: 10.1038/s41377-026-02270-0 (PMC13068960; doi:10.1038/s41377-026-02270-0)
Supplement: Supplementary file 1 — suplement [file 41377_2026_2270_MOESM1_ESM.pdf]

Supplementary Information for
Large-Area Metal-Integrated Grating Electrode Achieving Near 100% Infrared Transmission

Karolina Bogdanowicz,[1,2] Weronika Głowadzka,[1,2] Tristan Smołka,[3] Michał Rygała,[3] Marcin Kałuża,[4] Marek Ekielski,[1] Oskar Sadowski,[1,5] Magdalena Zadura,[1,2] Magdalena Marciniak,[2] Marcin Gębski,[2] Michał Wasiak,[2] Marcin Motyka,[3] Anna Szerling,[1] and Tomasz Czyszanowski[2,*]

[1]*Łukasiewicz Research Network – Institute of Microelectronics and Photonics, al. Lotników 32/46, 02-668 Warsaw, Poland*
[2]*Photonics Group, Institute of Physics, Lodz University of Technology, ul. Wólczańska 219, 90-924 Łódź, Poland*
[3]*Laboratory for Optical Spectroscopy of Nanostructures, Department of Experimental Physics, Faculty of Fundamental Problems of Technology, Wrocław University of Science and Technology, Wybrzeże Wyspiańskiego 27, 50-370 Wrocław, Poland*
[4]*Institute of Electronics, Lodz University of Technology, al. Politechniki 8, 93-590 Łódź, Poland*
[5]*Warsaw University of Technology – Institute of Microelectronics and Optoelectronics, 00-662 Warsaw, Koszykowa 75, Poland*

This PDF file includes:

- Supporting figures S1–S13
- Supporting tables S1–S4
- Supporting information: sections S1–S6

* tomasz.czyszanowski@p.lodz.pl

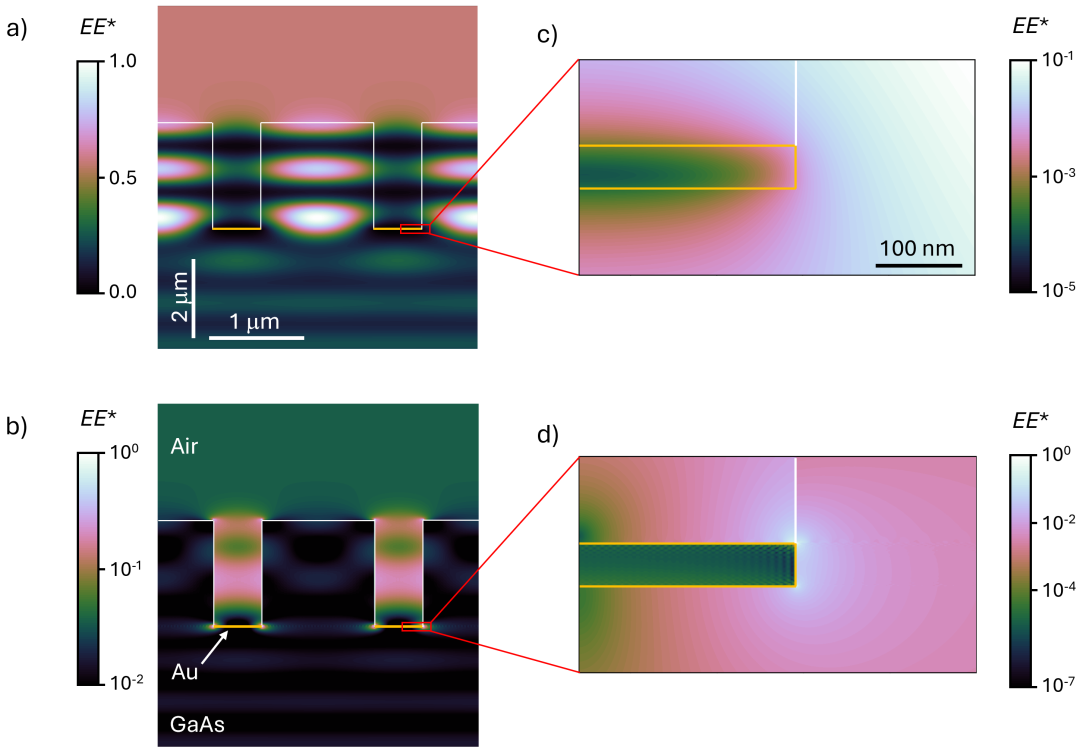

FIG. S1. Light intensity (EE^*) distribution under normal incidence from the substrate side in the case of a), c) TE and b), d) TM polarization in the yz-plane of the metalMHCG cross-section, c) and d) are zoomed distributions in the proximity of metal.

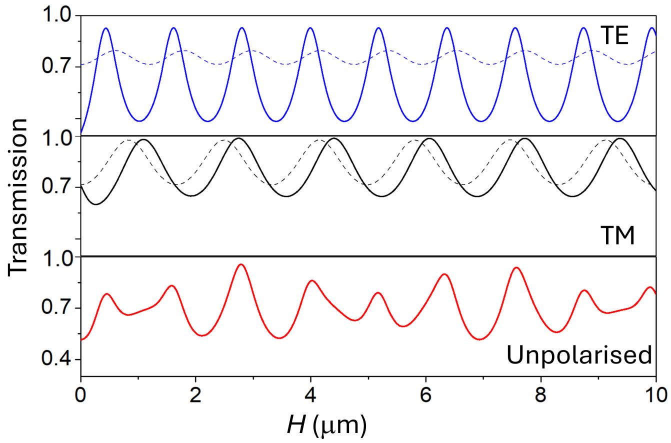

FIG. S2. Calculated transmission of both polarisations (solid lines blue and black) and unpolarized light (solid line red) as a function of semiconductor stripe height in the metalMHCG for $L = 1.4\,\mu\text{m}$, $F = 0.74$, $H_\text{m} = 50\,\text{nm}$ and a wavelength of $7\,\mu\text{m}$. Dashed lines represent transmission through semi-infinite GaAs with a homogeneous layer of height H and refractive index of $n_1 = 2.95$ (blue) and $n_2 = 2.11$ (black) on top. These indices correspond to the values of effective refractive indices calculated for TE and TM transmissions propagating through the metalMHCG (see Section II in the main text). Although the periodicity of transmission with respect to H is equal in both cases indicated by the same colors (metalMHCG and homogenous layer), the amplitudes of transmissions are significantly different, which is attributed to the presence of the metal in the metalMHCG case.

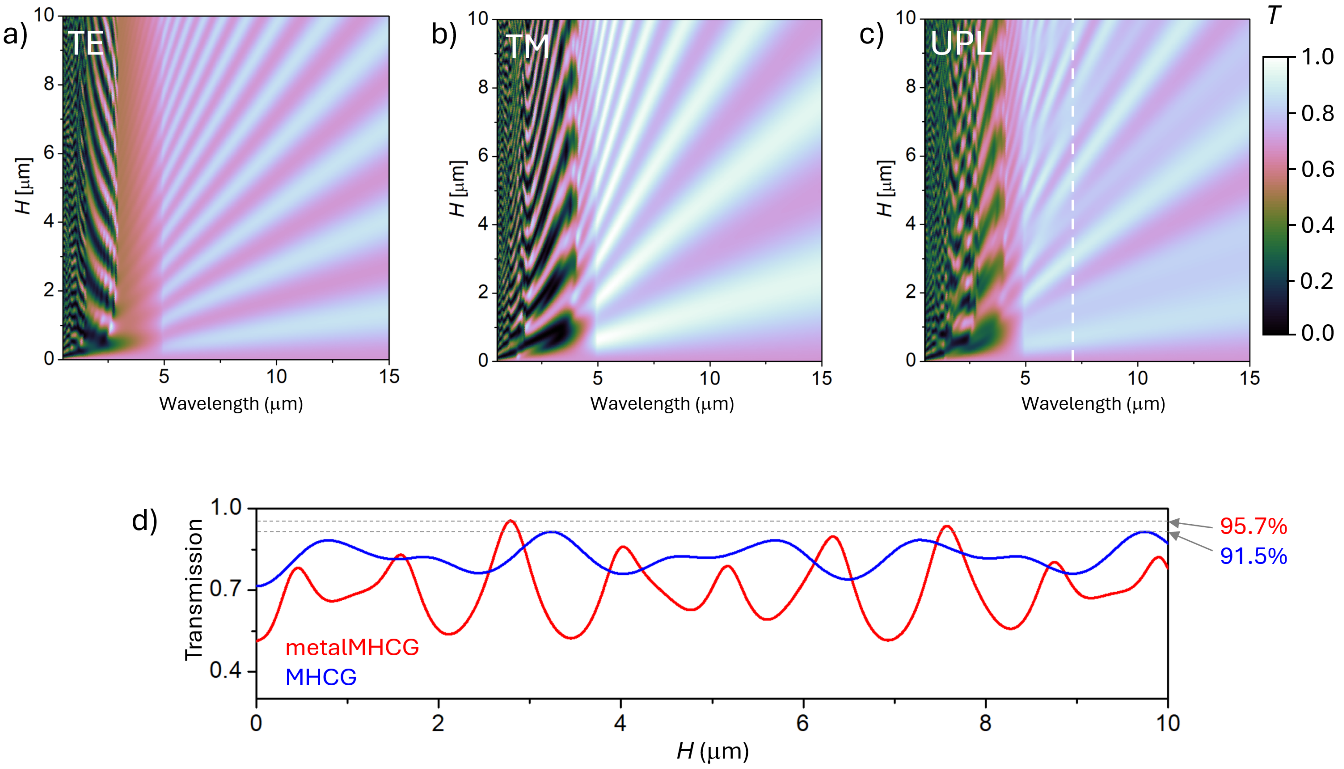

FIG. S3. Calculated transmission (T) maps of the MHCG (without gold) under normal incidence of a) TE, b) TM polarized light, c) unpolarized light in the domain of the wavelength, and height of the semiconductor stripes (H), the dashed white line indicates the wavelength used in d); d) unpolarized transmission as a function of the stripe height for the MHCG (blue) and metalMHCG (red). The paramaters of the MHCG and the metalMHCG are $L = 1.4\,\mu\text{m}$, $F = 0.56$ and $L = 1.4\,\mu\text{m}$, $F = 0.74$, respectively.

S1. OPTICAL PROPERTIES OF metalMHCG

A. Angular dependence

Figure S4a presents a top view of the metalMHCG structure, where the metallic and semiconductor stripes are indicated in orange and gray, respectively. Owing to the geometry of the metalMHCG, two orthogonal in-plane directions, x and y, can be defined as parallel and perpendicular to the stripes, respectively, allowing the corresponding in-plane wave-vector components k_x and k_y to be introduced. Figure S4(b) shows the transmission of unpolarized light incident on the metalMHCG in the (k_x, k_y) space. The transmission exhibits an isotropic character and approaches zero as $\sqrt{k_x^2 + k_y^2}$ approaches k_0, corresponding to an incidence angle of 90° in air and to the condition of total internal reflection for propagation from an optically denser medium. Figures S4c–f present the angular dependence of transmission as a function of the incidence angles with respect to the x and y axes, denoted as α_x and α_y, respectively, for both orthogonal polarizations as well as for unpolarized light, and for illumination from both the air side and the GaAs substrate side. The gray curves represent the transmission obtained for a GaAs surface coated with an ideal anti-reflection (AR) layer with a refractive index of $\sqrt{n_{\mathrm{GaAs}}}$, where n_{GaAs} is the refractive index of GaAs, and a thickness of $\lambda/(4n_{\mathrm{GaAs}})$, where λ is the wavelength. The results demonstrate that the metalMHCG exhibits transmission characteristics very close to those of an ideal AR coating, with only a slightly steeper roll-off near the limiting angles of incidence.

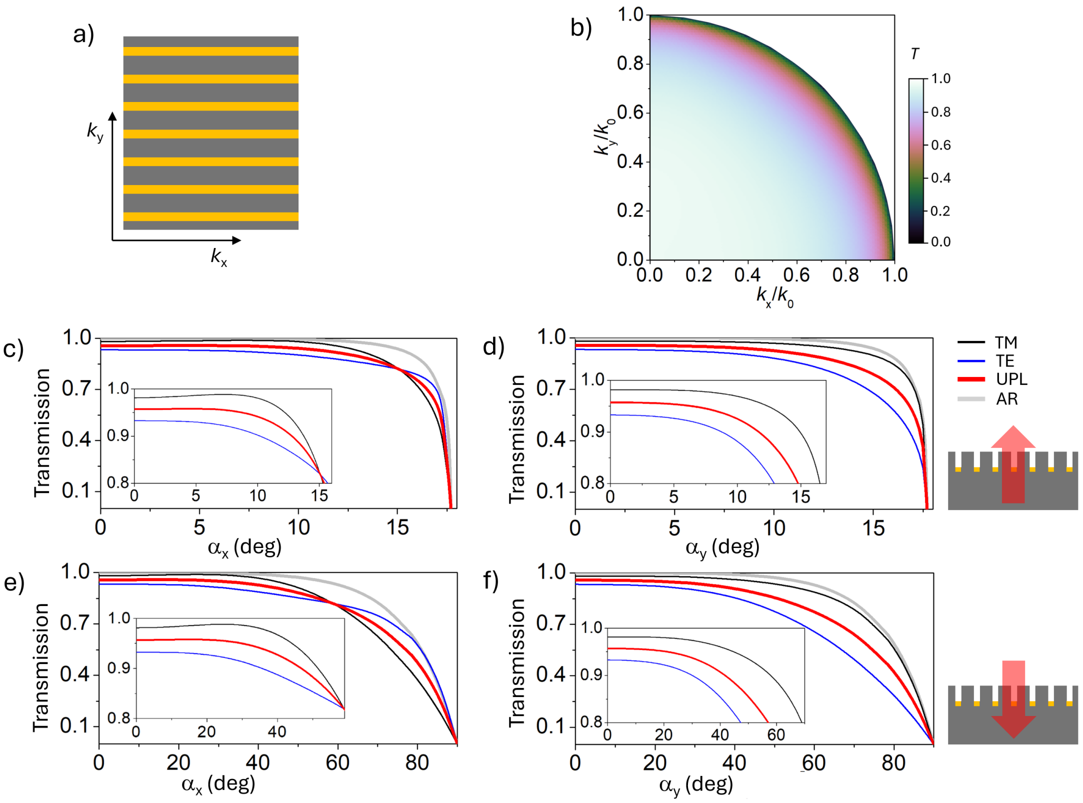

FIG. S4. a) Schematic top view of the metalMHCG geometry and the definition of the in-plane wave-vector components k_x and k_y. b) Calculated transmission T as a function of the normalized in-plane wave-vector components k_x/k_0 and k_y/k_0, where k_0 is the free-space wave number. Angular dependence of transmission for light incident from the GaAs substrate side as a function of the inclination angles α_x c) and α_y d), defined with respect to the x and y directions shown in panel a). Corresponding angular transmission characteristics for light incident from the air side as a function of the inclination angles α_x e) and α_y f). Black and blue curves represent transmission for TM- and TE-polarized light, respectively, while the red curves correspond to unpolarized light (UPL). The gray curves denote the transmission obtained with an ideal anti-reflection (AR) coating at the GaAs–air interface. Insets show enlarged views of the high-transmission angular ranges. The illumination geometries for substrate-side and air-side incidence are schematically illustrated next to the corresponding panels. All calculations were performed for metalMHCG parameters of $L = 1.43\,\mu\mathrm{m}$, $F = 0.74$, $H = 2.79\,\mu\mathrm{m}$, and $H_{\mathrm{m}} = 50\,\mathrm{nm}$ at a wavelength of $7\,\mu\mathrm{m}$.

B. Optimization of metalMHCG parameters

Identifying metalMHCG parameters that ensure maximum transmission of unpolarized light requires multidimensional optimization of the grating geometry. For metalMHCG structures with an effective refractive index corresponding to GaAs in the mid-infrared range, the highest transmission while maintaining a minimal stripe height H is achieved in the third transmission band (see Fig. 2(a) in the main text). The grating parameters L and H required to maximize transmission at a given wavelength must be scaled in a non-linear manner due to the dispersion of the refractive indices of both the semiconductor and the metal constituting the metalMHCG. Similarly, variations in the metal thickness H_m lead to non-linear changes in both L and H.

C. Metal thickness

Figures S5a,b show the grating period L and stripe height H that enable maximum transmission of unpolarized light through the metalMHCG (see Section S1 B) as functions of the gold stripe thickness. Figure S5c presents the corresponding sheet resistance of the metalMHCG, while Fig. S5d shows the polarized and unpolarized transmission. A structure without metallic stripes enables unpolarized-light transmission below 90%. The results indicate the existence of an optimal gold stripe thickness in the range of 50–100 nm, for which maximum unpolarized transmission is achieved. This behavior arises from a balance between a minimal amount of metal required to induce the anti-waveguiding effect, which suppresses light penetration into the metallic stripes, particularly for the TE component through the horizontal metal surfaces, and a sufficiently small thickness to reduce penetration of the TM component into the metal volume via plasmonic effects at the sidewalls of the gold stripes.

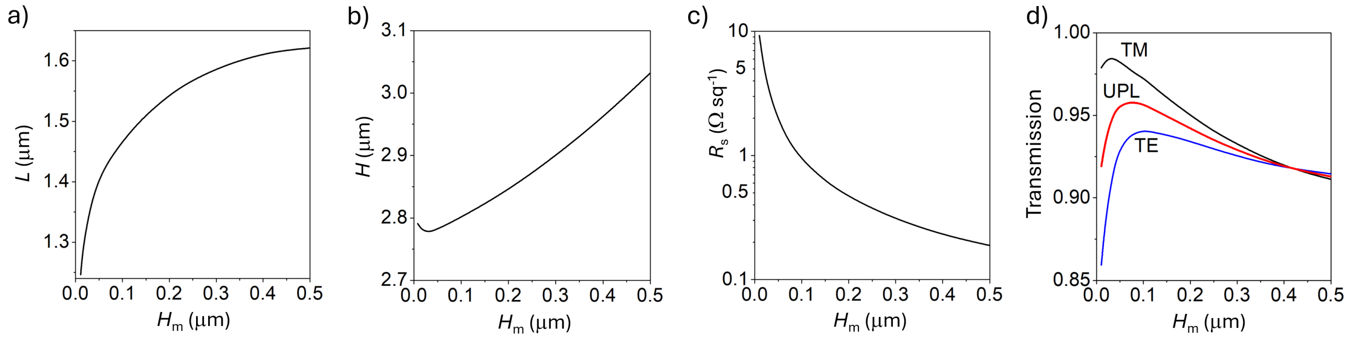

FIG. S5. Optimal parameters of the grating ensuring maximum transmission as a function of the metal stripe thickness H_m. a) Optimized grating period L and b) optimized height of the semiconductor stripes H. c) Corresponding sheet resistance R_s of the metalMHCG. d) Calculated transmission for TM-polarized (black), TE-polarized (blue), and unpolarized (UPL, red) light. Each data point corresponds to the result of a numerical optimization in which the grating period L and the semiconductor stripe height H were optimized for a fixed fill factor $F = 0.74$.

D. Transmission wavelength tunning

In Fig. S6a the curve labeled H_{const} shows the maximum transmission through the metalMHCG as a function of wavelength for a fixed semiconductor stripe height $H = 2.79\,\mu\text{m}$ and metal thickness $H_m = 50\,\text{nm}$. This configuration corresponds to a practical scenario in which gratings with different transverse dimensions (L, F) are fabricated on a single wafer in a single processing run. The burgundy curve labeled H_{var} shows the maximum transmission as a function of wavelength when the parameters L, F, and H are optimized simultaneously (see Section S1 B), while the metal thickness remains fixed at $H_m = 50\,\text{nm}$. For the H_{const} case, the strong variation of the maximum transmission results from the fact that a fixed value of H corresponds to successive transmission bands as the wavelength decreases, starting from the first transmission band at the longest wavelengths. Over the entire analyzed spectral range, the transmission values for the H_{const} configuration remain above the Fresnel limit.

Figures S6b,c illustrate the transmission spectra of the metalMHCG for different central wavelengths in the H_{const} case. Figure S6b shows spectra corresponding to different optimized configurations whose transmission maxima are located near 7 μm, whereas the spectra shown in Fig. S6c correspond to successive maxima of the H_{const} curve in Fig. S6a. The spectra associated with the longest wavelengths exhibit noticeably lower peak transmission values and

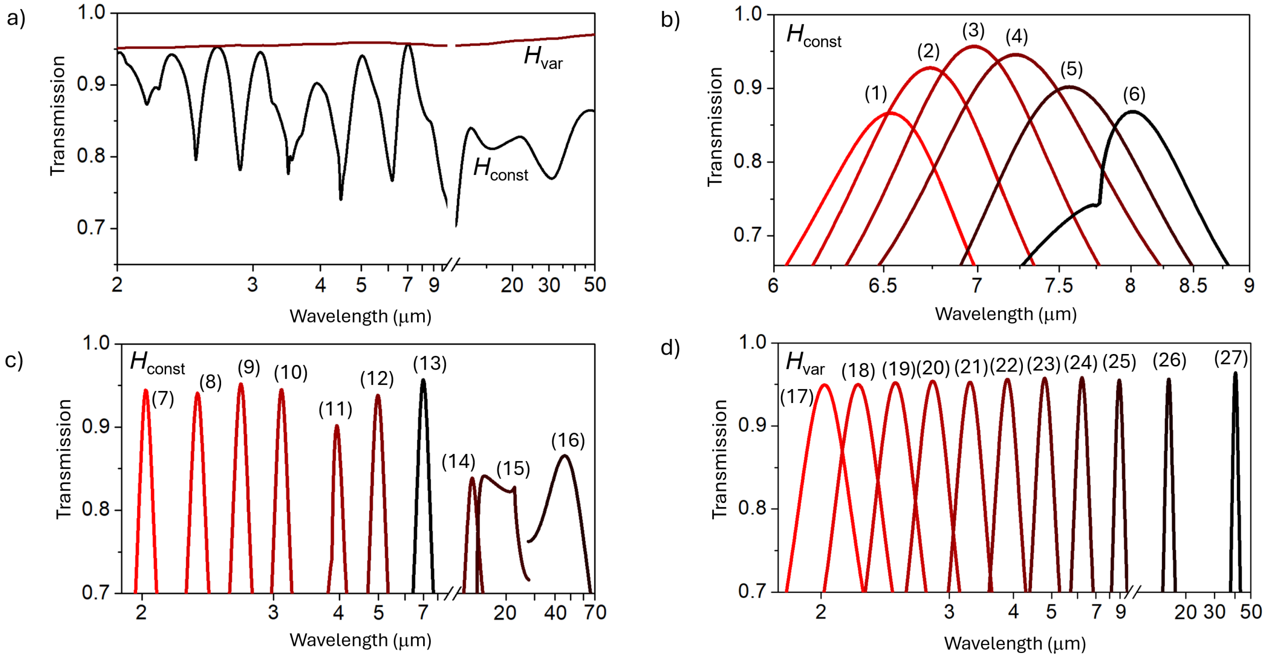

FIG. S6. Transmission characteristics of the metalMHCG. a) Maximum transmission as a function of wavelength obtained for two optimization scenarios: a fixed height of the semiconductor stripes (H_{const}), where only the grating period L and fill factor F are optimized, and a variable stripe height (H_{var}), where L, F, and H are optimized simultaneously. b)–d) Representative transmission spectra for metalMHCGs designed at selected central wavelengths. b) Transmission spectra for the H_{const} case in the vicinity of $\lambda = 7\,\mu\text{m}$. c) Transmission spectra corresponding to the transmission maxima shown in a) for the H_{const} case. d) Transmission spectra at selected wavelengths for the H_{var} case. The numbers in parentheses denote the configurations whose central wavelengths, geometric parameters, and numerical values of the maximum unpolarized transmission are summarized in Tab. S1.

broader spectral widths, which is a characteristic feature of the first and second transmission bands. As shown in Fig. 2a of the main text and Fig. S3c, these bands exhibit the smallest slopes of the high-transmission regions with respect to wavelength axis, resulting in their broad spectral response.

Figure S6d presents transmission spectra for selected wavelengths in the H_{var} case. The maximum transmission slightly increases, while the relative spectral bandwidth decreases with increasing wavelength, primarily due to the increasing magnitude of the complex refractive index of gold. The numbers in parentheses denote the configurations whose spectra are shown in Figs. S6b–d, while the corresponding central wavelengths, geometric parameters, and numerical values of the maximum unpolarized transmission are summarized in Tab. S1. The results collected in Fig. S6 demonstrate that the metalMHCG enables near-unity transmission over a very broad infrared spectral range, as well as in the visible range, as reported in previous studies [1]. The metalMHCG structure enables transmission significantly exceeding the Fresnel limit over a wide wavelength range by effectively reducing the interaction of the optical field with free electrons in the metal.

TABLE S1. Optimized metalMHCG geometric parameters and corresponding transmission values for selected wavelengths indicated by the numbers in Fig. S6. Wavelength is denoted by λ, L is the grating period, F the fill factor, and H the height of the semiconductor grating stripes. UPL corresponds to the maximum transmission of unpolarized light, while TE and TM denote the transmission values for TE- and TM-polarized light, respectively, at the wavelength corresponding to the maximum UPL.

No.	λ [nm]	L [µm]	F	H [µm]	TE	TM	UPL
1	6390	1.24	0.647	2.79	0.793	0.880	0.836
2	6660	1.35	0.682	2.79	0.901	0.940	0.920
3	7000	1.43	0.734	2.79	0.931	0.982	0.957
4	7290	1.47	0.780	2.79	0.903	0.981	0.942
5	7650	2.04	0.721	2.79	0.823	0.973	0.898
6	8040	2.37	0.706	2.79	0.769	0.967	0.868
7	2019	0.232	0.805	2.79	0.901	0.989	0.945
8	2329	0.489	0.806	2.79	0.895	0.986	0.941
9	2668	0.472	0.806	2.79	0.914	0.991	0.952
10	3092	0.461	0.774	2.79	0.910	0.981	0.946
11	3956	1.17	0.754	2.79	0.841	0.963	0.902
12	4965	1.29	0.731	2.79	0.895	0.981	0.938
13	6963	1.37	0.738	2.79	0.931	0.982	0.957
14	12340	2.39	0.772	2.79	0.922	0.754	0.838
15	22690	7.03	0.730	2.79	0.670	0.983	0.826
16	46280	7.46	0.864	2.79	0.944	0.787	0.865

No.	λ [nm]	L [µm]	F	H [µm]	TE	TM	UPL
17	2020	0.360	0.790	0.877	0.917	0.986	0.951
18	2210	0.418	0.780	0.972	0.921	0.983	0.952
19	2480	0.469	0.780	1.09	0.922	0.986	0.954
20	2820	0.538	0.780	1.23	0.923	0.988	0.955
21	3270	0.700	0.748	1.44	0.931	0.979	0.955
22	3960	0.848	0.747	1.74	0.932	0.982	0.957
23	4780	1.03	0.747	2.09	0.933	0.984	0.959
24	6230	1.26	0.737	2.48	0.935	0.982	0.958
25	8920	1.81	0.739	3.55	0.927	0.983	0.955
26	15710	3.71	0.714	6.85	0.934	0.981	0.958
27	40240	8.64	0.723	16.6	0.955	0.982	0.968

S2. FABRICATION UNIFORMITY

To quantify fabrication-related variability, we performed a statistical analysis of the grating period L and the width of the semiconductor stripes a. The parameters were extracted from scanning electron microscopy (SEM) images acquired from nine metalMHCG regions, numbered as shown in Fig. S7, each encompassing approximately 15 grating periods (see Fig. S8). For each parameter $x = L, a, H, H_m$, we determined its relative standard deviation (RSD), defined as the ratio of the standard deviation (SD) of x to the mean value $\bar{x}$ and expressed as a percentage.

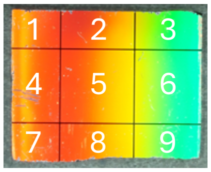

FIG. S7. Image of the sample with numbered regions indicating the areas in which statistical analysis of the grating period L and the stripe width a was performed and summarized in Tab. S2.

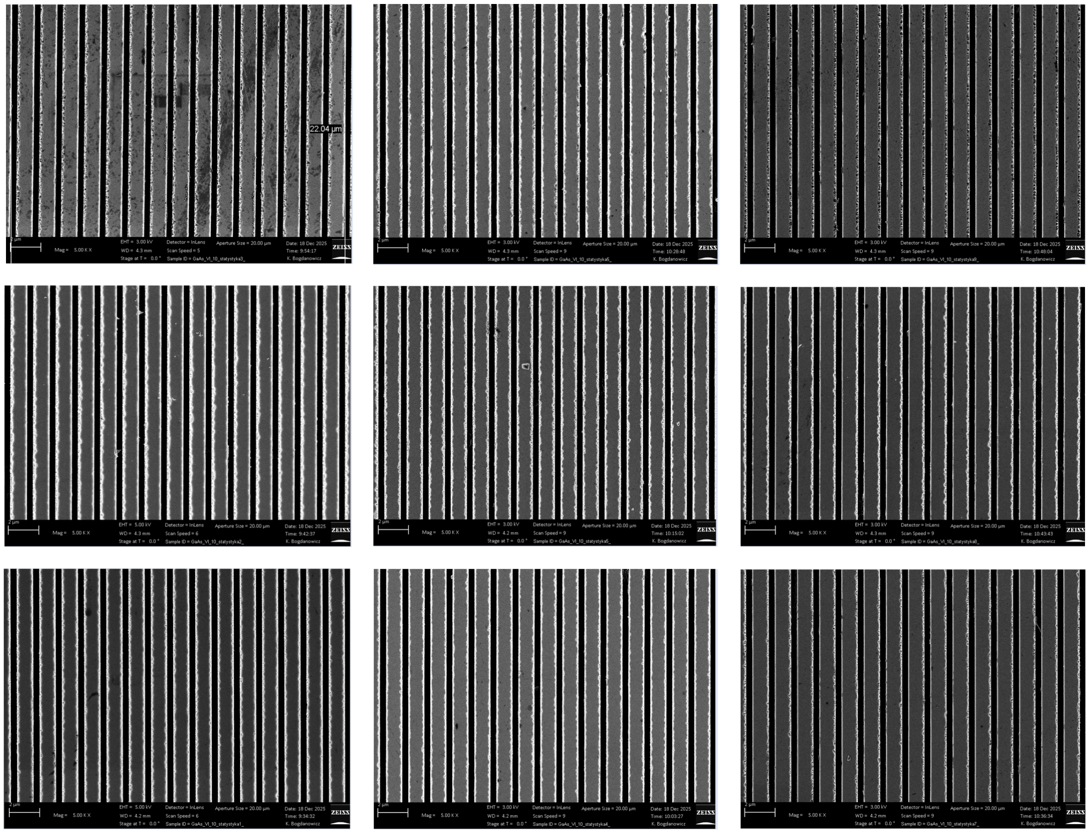

FIG. S8. Exemplary top-view SEM images of metalMHCG used for the statistical analysis, acquired in nine sample regions. The arrangement of the images corresponds to the layout of the regions shown in Fig. S7.

The vertical dimensions (H and H_m) were determined from a focused ion beam (FIB) cross-section prepared on a reference sample fabricated under identical processing conditions. The mean value and relative standard deviation (RSD) of all metalMHCG geometrical parameters are summarized in Tab. S2. Across the entire sample, the relative standard deviation of the grating period, RSD(L), was at the level of 0.1%, corresponding to an absolute standard deviation of only a few nanometres. For the semiconductor stripe width a, relative standard deviation RSD(a) below 1% was observed across the structure, with the only exception of region 1, where RSD(a) reached 1.3%. Such low variability of L arises from the fact that the grating period is defined macroscopically by the total patterned width of the structure and its uniform subdivision into an integer number of periods during lithography, rather than by the local definition of individual features. Experimentally, L is determined by measuring the cumulative width of

TABLE S2. Mean values (denoted by overbars) and relative standard deviations (RSDs) of the grating period L and the semiconductor stripe width a, determined for the regions numbered as indicated in Fig. S7, as well as the mean values and RSDs of H and H_m determined on a reference sample fabricated under identical processing conditions.

Region	$\bar{L}$ [nm]	RSD(L)	$\bar{a}$ [nm]	RSD(a)	$\bar{H}$ [nm]	RSD(H)	$\bar{H}_m$ [nm]	RSD(H_m)
1	1472	0.1%	1076	1.3%	2890	1.4%	51	3.9%
2	1467	0.1%	1094	0.5%				
3	1469	0.1%	1107	0.6%				
4	1467	0.1%	1084	0.5%				
5	1470	0.1%	1091	0.6%				
6	1469	0.1%	1101	0.6%				
7	1471	0.1%	1082	0.3%				
8	1469	0.1%	1082	0.3%				
9	1469	0.1%	1086	0.4%				

multiple grating periods and dividing it by the number of periods—15 periods in the present analysis. This averaging procedure reduces the measurement uncertainty compared to parameters extracted from individual features, such as the width of a single semiconductor stripe a, which are more sensitive to local variations and edge roughness. Numerical simulations indicate that deviations of the magnitude indicated in Tab. S2 for the parameters L, a, and H_m result in only a negligible reduction in transmission. The transmission is more sensitive to variations in the parameter H, for which a relative standard deviation of 1.4% may result in a transmission reduction of approximately 1.5%. Together with deviations from an ideal rectangular cross-sectional profile, these fabrication-induced variations may account for the discrepancy between experimental results and numerical simulations. Overall, the observed relative deviations indicate good fabrication uniformity across the sample.

S3. MEASUREMENT SETUP

Transmittance measurements were conducted using a Vertex 80v vacuum Fourier Transform Infrared spectrometer (FTIR) from Bruker. Due to the many limitations and difficulties of working in the mid-infrared spectral region, it was necessary to use a Fourier spectrometer instead of a simpler monochromator-based setup. The advantages of using the FTIR approach over the dispersive setup have been extensively studied and described [2–5]. Figure S9a shows the experimental setup for transmittance measurements. The sample light generated by a polychromatic source (either a halogen or a glow bar, depending on the spectral range) was guided by parabolic golden mirrors to the Michelson interferometer and then focused onto the sample at a normal incident angle, creating a roughly 1-mm diameter spot entirely contained within the metalMHCG sample. The sample was placed on a mounting holder with a slit slightly larger than the diameter of the focused spot, which allowed light to transmit through the entire sample and exit from the etched grating. The transmitted light was then directed to a HgCdTe (MCT) liquid-nitrogen cooled detector and transferred as an electrical response into an analog-to-digital converter. The signal was later modified using a 3-Term Blackman-Harris apodization function or an adequate zero-filling factor and converted into a spectrum using Fast Fourier Transform. To perform polarization-resolved measurements, a KRS-5 wire-grid polarizer was inserted into the beam path before the light reached the sample, as shown in Fig. S9b. Using this experimental procedure, we were able to obtain spectra in the 1.3–15 µm spectral range with a 4 cm^{-1} resolution. To ensure measurement repeatability, the FTIR spectrometer was calibrated daily in accordance with the manufacturer's guidelines, with automatic Michelson interferometer calibration performed during software initialization. Source power stability and detector response were verified by comparing reference spectra acquired without a sample at different times during the day and across consecutive measurement days. The detector temperature remained stable for approximately 24 hours after filling the dewar with liquid nitrogen and was continuously monitored by the spectrometer software. Wavelength calibration was performed by matching spectral features acquired with a non-vacuumed sample compartment to atmospheric absorption lines from reference databases. The measured transmittance of the GaAs substrate used as a reference was 55%, in agreement with literature values.

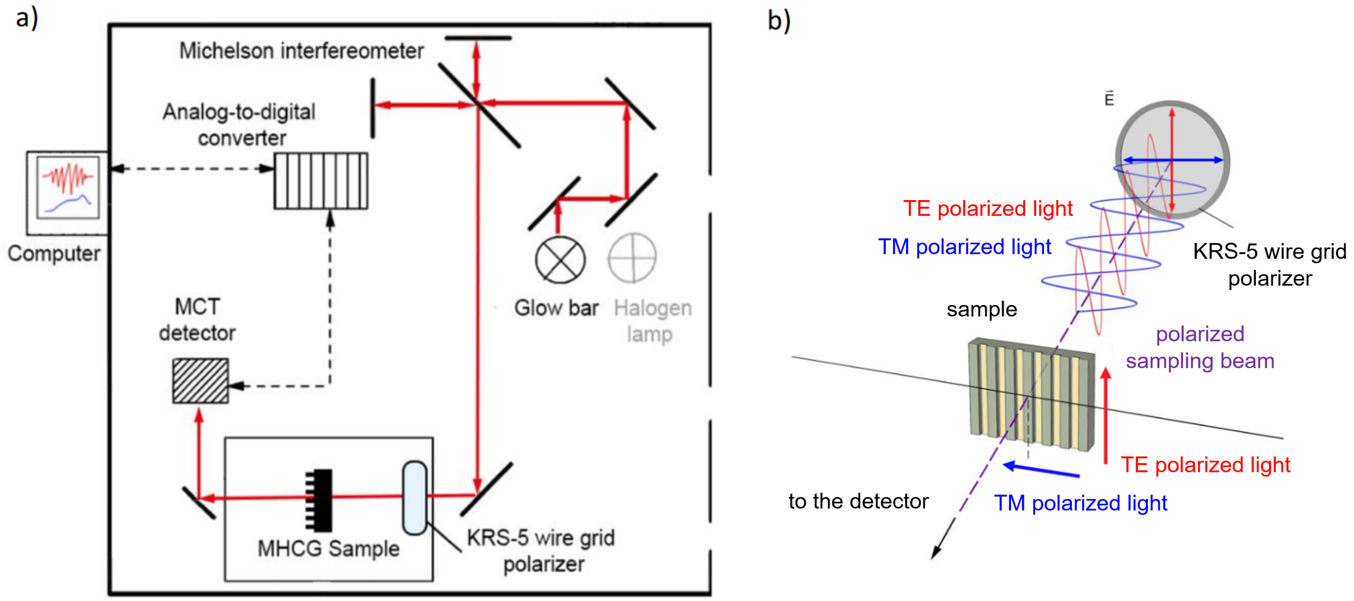

FIG. S9. a) Schematic view of the beam path in a transmittance experiment with FTIR setup; b) close-up of polarizer mounting showing the direction of stripes in the sample

S4. COMPARISON OF EXPERIMENTAL AND NUMERICAL SPECTRA FOR THE ACTUAL metalMHCG CROSS-SECTION

To compare the experimental spectrum with the numerically computed spectrum (Fig. S10a), we implemented the metalMHCG cross-section derived from the SEM image (Fig. S10b), as seen in Fig. S10c, in PLaSK software [6], which was used for all calculations in this study. In Fig. S10a, the experimentally measured spectrum (black line) is shown alongside three numerically computed spectra. The green dashed line corresponds to the spectrum calculated using

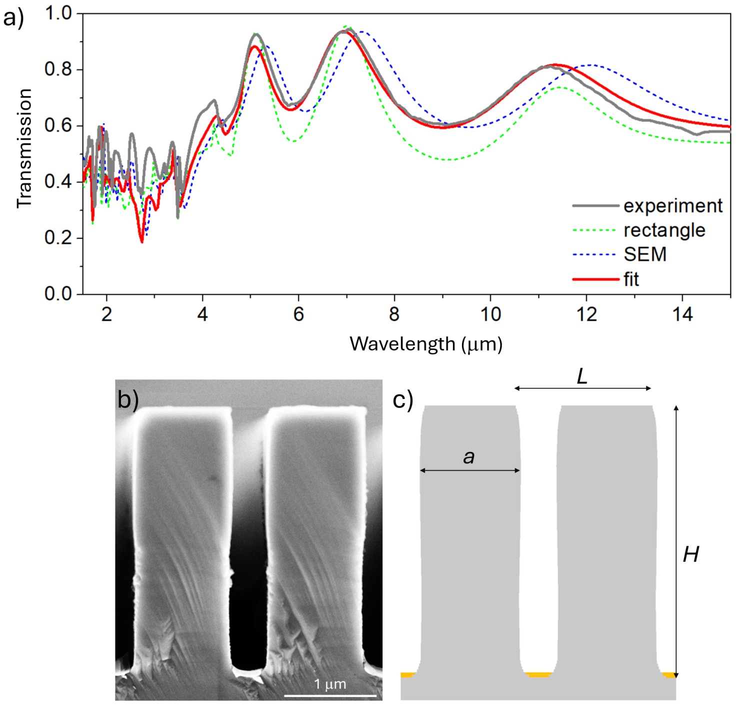

FIG. S10. a) Experimental (black line) and calculated (other lines) spectra of the metalMHCG with geometrical parameters defined in Tab. S3; b) cross-section scanning electron microscope (SEM) image of metalMHCG; c) image of the metalMHCG implemented in the numerical algorithm.

the optimal parameters for a rectangular metalMHCG cross-section (rectangular), with the parameters defined in Tab. S3 (first row). The blue dashed line represents the spectrum computed based on the cross-section and geometric parameters (SEM), extracted from the SEM image (see Tab. S3, second row). The red solid curve represents the spectrum obtained by optimization using the SEM-derived cross-section and metalMHCG parameters (L, F, H) to minimize the sum of squared differences between the experimental and computed spectra (first and third rows in Tab. S3).

All curves are closely aligned, accurately identifying both the positions and values of the transmission maxima and minima. The red curve, resulting from the optimization procedure, exhibits the closest match to the experimental data. By comparing the metalMHCG parameters in Tab. S3, it can be observed that the height (H) extracted from the SEM image differs noticeably from the value obtained through the optimization procedure. The SEM-based height measurement may be inaccurate due to charging effects at the surface of undoped GaAs. As a result, the height (2.847 μm) derived from SEM image may be overestimated, as suggested by the result of optimization procedure, where the height H is determined to be 2.7 μm.

TABLE S3. Geometrical parameters of the metalMHCG for various cross-section shapes.

	L [μm]	$F = a/L$	H [μm]	Cross-section
rectangle	1.431	0.734	2.787	Rectangle
SEM	1.470	0.759	2.847	SEM image
fit	1.465	0.745	2.699	SEM image

S5. MEASUREMENT UNCERTAINTY

A series of position-dependent measurements was performed (see Fig. S11a) to evaluate the transmission uniformity in region 5 of the sample. For measurements conducted with a 1 mm spot size (see Fig. S11a and blue circles), spatially limited by the physical aperture in the FTIR setup, a deviation of ±1% was observed, as illustrated in Fig. S11b. Similar position-dependent measurements were repeated at a larger number of positions using a smaller spot defined by a 0.25 mm aperture (see Fig. S11a and yellow circles), which is the smallest aperture available in the experimental setup. In this case, a slightly higher total deviation of 1.5% was obtained, as illustrated in Fig. S11c. It should be noted that, due to experimental limitations, the sample was slightly out of focus, resulting in a probed area approximately twice as large as the aperture, while remaining smaller than the overall sample dimensions. All measurements presented in the main text were performed using a 1 mm aperture, chosen to maximize the effective collection efficiency of the detector. Using larger apertures results in partial clipping of the transmitted beam by the detector active area, thereby increasing the measurement uncertainty. To assess measurement reproducibility, datasets acquired on different days were compared, and the measured transmission spectra were found to remain within the uncertainty range determined from the position-dependent measurements. It should be noted that the measurements presented in this section, which show a maximum transmission reduced by approximately 1.5% relative to the maximum value reported in the main text, were performed one year after the measurements reported in the main text. During this period, the sample was subjected to numerous measurements described in this work, as well as to additional experiments not related to the present study. The measurements presented here, reflecting spatial uncertainty, therefore also indirectly indicate the degree of metalMHCG degradation resulting from cumulative mechanical handling.

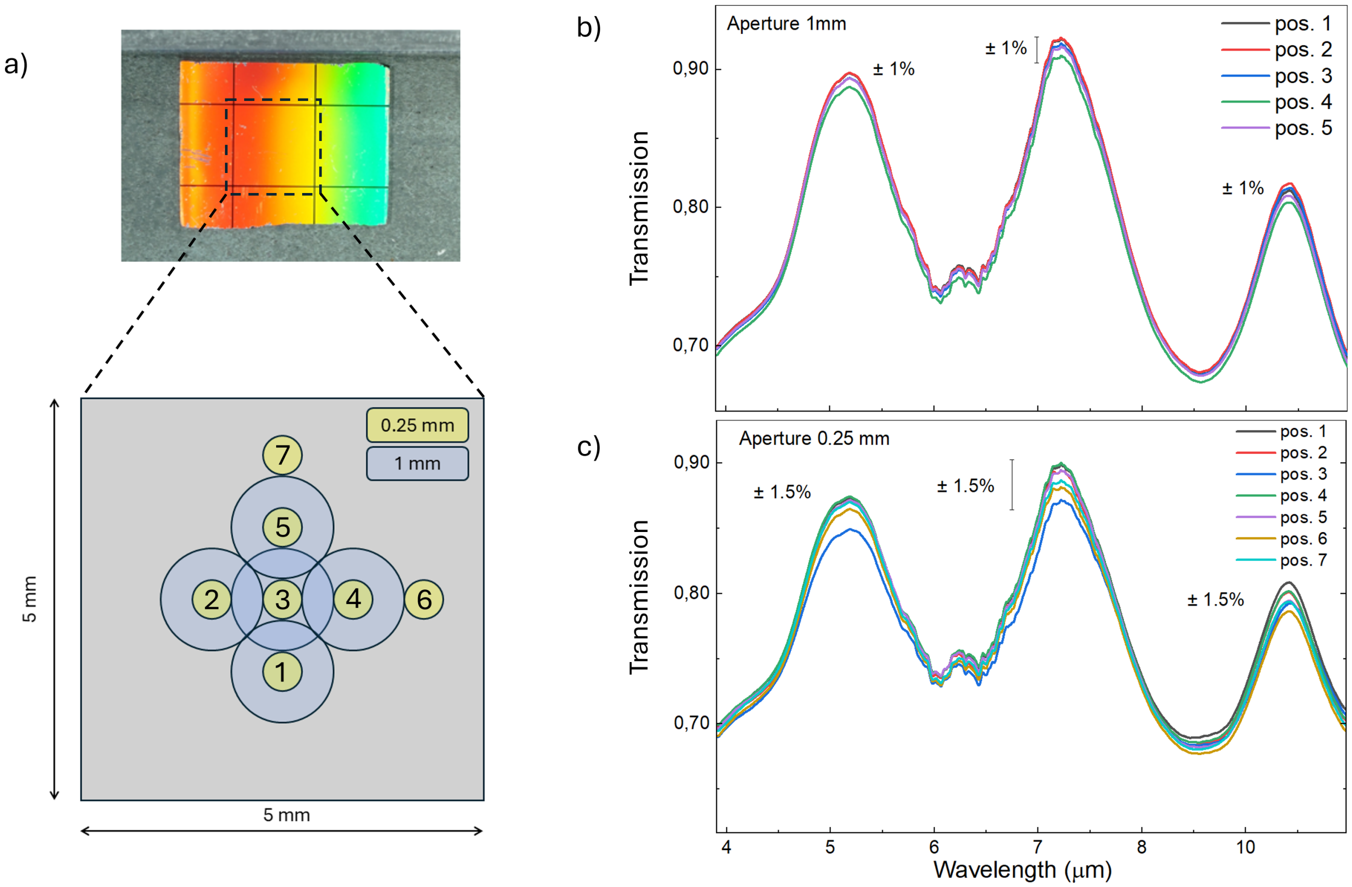

FIG. S11. a) Image of the sample with schematically illustrated approximate measurement positions labeled from 1 to 5 for a 1 mm aperture and from 1 to 7 for a 0.25 mm aperture. b) Transmission spectra measured at different spot positions for a 1 mm aperture. c) Transmission spectra measured at different spot positions for a 0.25 mm aperture.

S6. INFRARED PHOTOGRAPHY

To compare the transmission through the GaAs substrate and the GaAs substrate with metalMHCG, a thermal imaging experiment was carried out. A QR code was manufactured on a FR4 PCB laminate, providing a strong emissivity contrast to the copper-made code. The dimensions of the code were chosen taking into account the minimum focusing distance of the thermographic camera with the lens used and the limits imposed by its instantaneous field of view (IFOV) parameter. The QR code was placed on a heatbed and imaged using a FLIR X6901SC InSb cooled thermographic camera operating in the 3–5 µm band. The camera was equipped with an extension ring and a 4.8–5.0 µm band-pass optical filter. The spectral characteristics of the optical filter in the proximity of fourth transmission band are illustrated in Fig. S12a. The 1/4 inch ring was used for magnification, short enough to avoid image vignetting. As the filter considerably reduced the energy reaching the camera detector matrix, the code was heated to the temperature of 80°C using the heatbed and the camera integration time was adjusted. Heating the QR code increased the thermal contrast between the copper features and the exposed FR4 substrate, thereby improving the visibility of the transmitted image. The temperature of 80 °C was chosen as a compromise between sufficient radiative emission and safe operating conditions, remaining below the thermal limits of the 3D-printed components used in the experimental setup. The metalMHCG sample was not intentionally heated during the experiment. The GaAs substrate and the GaAs substrate with metalMHCG were placed in a dedicated 3D printed sample holder with a narrow slot, allowing for easy movement of both samples above the QR code and subsequent acquisition of the code thermograms through both samples, column by column. A series of 9 thermograms was taken and the fragments of the thermograms obtained through the slot were merged together, obtaining a final thermogram of the complete QR code. A picture of the code is shown in Fig. S12b and an image of the sample holder with both samples and the code visible beneath is shown in Fig. S12c. The complete test setup is shown in Fig. S12d.

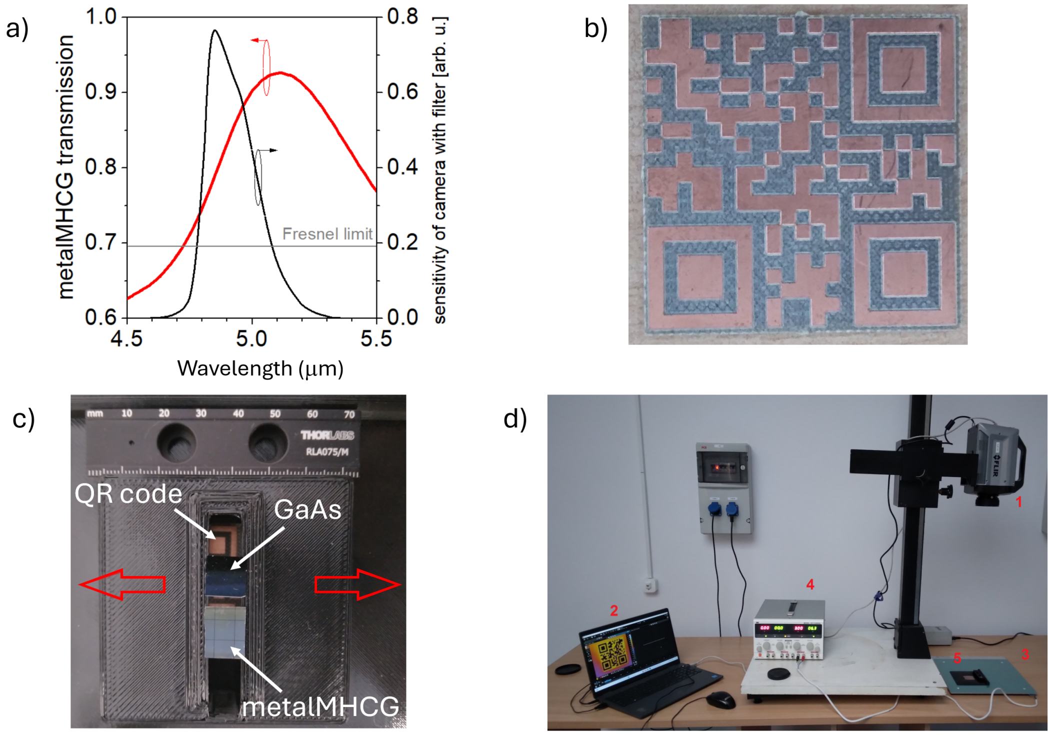

FIG. S12. a) Transmission spectrum in the proximity of fourth transmission band (red line, left axis) and spectral sensitivity of the camera with filter (black line, right axis), b) visible light picture of the 21 × 21 QR code fabricated on FR4 PCB laminate used for thermographic imaging; c) the holder with GaAs sample (top) and metalMHCG sample (bottom) and the code visible underneath through the slot, d) the test setup used for thermographic visualisation: 1. thermographic camera; 2. camera control computer; 3. heatbed; 4. heatbed DC power supply; 5. QR code.

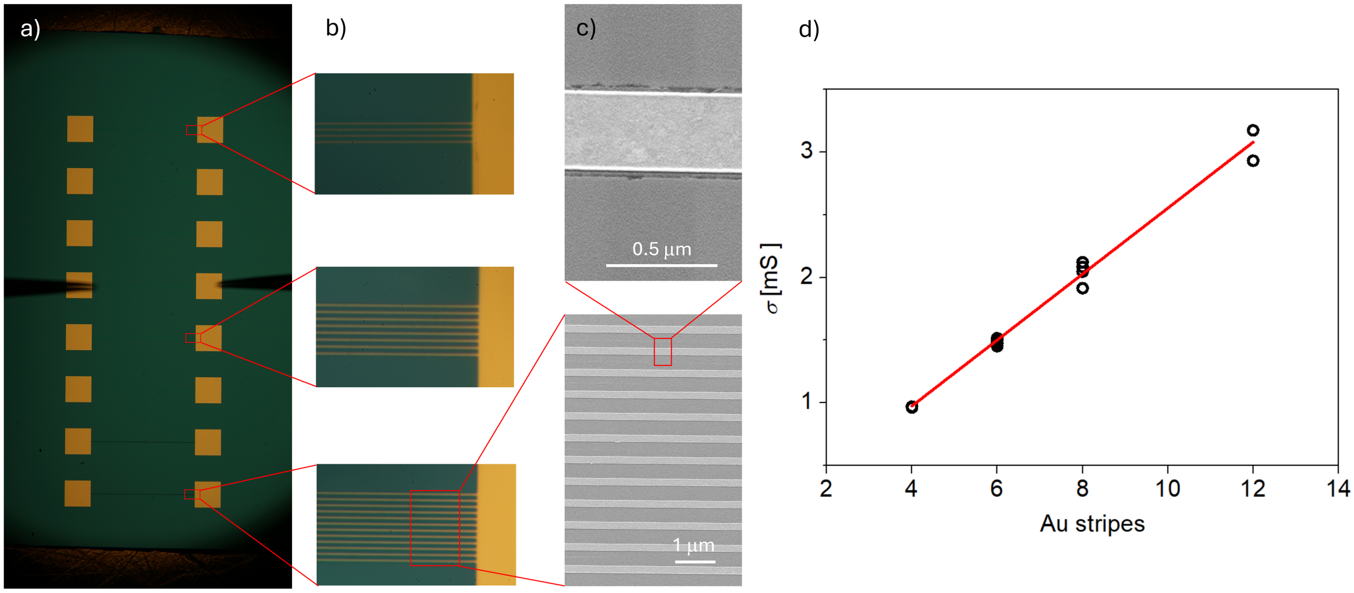

FIG. S13. Visible microscope images of (a) gold pads and (b) 50 nm-thick gold wires, along with their (c) corresponding SEM images, intended for electrical conductivity characterization; d) conductance measured for different number of gold stripes.

TABLE S4. Reference data used to generate Fig. 5 in the main text. The listed references correspond to the articles cited in the main text. The subsequent columns report the material used for the TCE (or structure), the transmission T through the TCE alone as reported in the corresponding article, and the Fresnel-limit transmission T_{Fr} calculated from the refractive index of the substrate material reported in the article, the calculated transmission through the TCE deposited on GaAs T_{GaAs}, and the sheet resistance reported in the article, R_s, [Ω/sq].

Ref.	TCE	t	T_{Fr}	T_{GaAs}	R_s [Ω/sq]
[31]	SnO_2	0.80	0.97	0.59	$1 \cdot 10^3$
[32]	SnO_2:W	0.80	0.94	0.61	400
[33]	SnO_2:Sb	0.50	0.97	0.37	$6.7 \cdot 10^4$
[34]	SnO_2:Co	0.60	0.94	0.46	51
[35]	In_2O_3	0.55	0.97	0.40	3.9
[36]	In_2O_3:Sn	0.61	0.94	0.46	61.2
[37]	ZnO	0.80	0.90	0.63	28.5
[38]	ZnO:Al	0.60	0.97	0.44	125
[39]	Y_2O_3:Ru	0.70	0.96	0.52	$2.1 \cdot 10^5$
[40]	$BaSnO_3$	0.75	0.86	0.62	$5 \cdot 10^3$
	$BaSnO_3$:La	0.45	0.86	0.37	196
[41]	Mg–C–O–H	0.70	0.97	0.51	108
[42]	$CuScO_2$	0.85	0.94	0.65	$4.36 \cdot 10^4$
[43]	$CuScO_2$:Sn	0.90	0.94	0.69	$5.2 \cdot 10^4$
	$CuScO_2$:Sn	0.85	0.94	0.65	$9.49 \cdot 10^3$
	$CuScO_2$:Sn	0.82	0.94	0.63	$2.8 \cdot 10^3$
[44]	$CuAlO_2$	0.70	0.96	0.52	206
[45]	CNT	0.83	0.96	0.62	50
	CNT	0.70	0.96	0.52	17
	CNT	0.72	0.96	0.54	10
	CNT	0.65	0.96	0.48	5

Ref.	TCE	T	T_{Fr}	T_{GaAs}	R_s [Ω/sq]
[46]	Graphene	0.88	0.94	0.67	$2.5 \cdot 10^4$
	Graphene	0.76	0.94	0.58	$1.98 \cdot 10^4$
	Graphene	0.74	0.94	0.56	$1.6 \cdot 10^3$
	Graphene	0.71	0.94	0.54	$1.31 \cdot 10^3$
[47]	Cr	0.82	0.96	0.61	$7.5 \cdot 10^3$
	Cr	0.73	0.96	0.54	600
	Cr	0.55	0.96	0.41	300
	Ni	0.79	0.96	0.59	$1 \cdot 10^3$
	Ni	0.65	0.96	0.48	290
	Ni	0.63	0.96	0.47	100
	Ni	0.45	0.96	0.33	30
[48]	$BaCu_2S_2$	0.45	0.96	0.34	$1.37 \cdot 10^3$
[49]	InGaBiAs:Si	0.72	0.96	0.54	7.05
[50]	$LaSe_2$	0.70	0.94	0.53	$2.25 \cdot 10^4$
[51]	BaCuSF	0.40	0.97	0.29	417
[21]	metalMHCG	–	0.72	0.78	1.08
[21]	metalMHCG	–	0.72	0.76	0.53
This work	metalMHCG	–	0.72	0.94	2.8

[1] Sokol, A. K. & Czyszanowski, T. Nearly perfect transmission of unpolarized infrared radiation through a one-dimensional metal grating embedded in a monolithic high-contrast grating. *Optics Express* **28** (2020).

[2] Thermofisher.com. URL https://assets.thermofisher.com/TFS-Assets/LSG/Application-Notes/TN50674-E-0215M-FT-IR-Advantages.pdf. Accessed: 2023-07-21.

[3] Griffiths, P. R. & A., D. H. J. *Fourier transform infrared spectrometry* (Wiley, 2007).

[4] Motyka, M. & Misiewicz, J. Fast differential reflectance spectroscopy of semiconductor structures for infrared applications by using fourier transform spectrometer. *Applied Physics Express* **3** (2010).

[5] Motyka, M. *et al.* Fourier-transformed photoreflectance and fast differential reflectance of hgcdte layers. the issues of spectral resolution and fabry–perot oscillations. *Measurement Science and Technology* **22**, 125601 (2011).

[6] Plask.com. URL https://plask.app/. Accessed: 2026-06-08.
